# Chromosome-level genome assembly, annotation and evolutionary analysis of the ornamental plant *Asparagus setaceus*

**DOI:** 10.1038/s41438-020-0271-y

**Published:** 2020-04-01

**Authors:** Shu-Fen Li, Jin Wang, Ran Dong, Hong-Wei Zhu, Li-Na Lan, Yu-Lan Zhang, Ning Li, Chuan-Liang Deng, Wu-Jun Gao

**Affiliations:** College of Life Sciences, Henan Normal University, Xinxiang, 453007 China

**Keywords:** Plant evolution, Plant molecular biology

## Abstract

*Asparagus setaceus* is a popular ornamental plant cultivated in tropical and subtropical regions globally. Here, we constructed a chromosome-scale reference genome of *A. setaceus* to facilitate the investigation of its genome characteristics and evolution. Using a combination of Nanopore long reads, Illumina short reads, 10× Genomics linked reads, and Hi-C data, we generated a high-quality genome assembly of *A. setaceus* covering 710.15 Mb, accounting for 98.63% of the estimated genome size. A total of 96.85% of the sequences were anchored to ten superscaffolds corresponding to the ten chromosomes. The genome of *A. setaceus* was predicted to contain 28,410 genes, 25,649 (90.28%) of which were functionally annotated. A total of 65.59% of the genome was occupied by repetitive sequences, among which long terminal repeats were predominant (42.51% of the whole genome). Evolutionary analysis revealed an estimated divergence time of *A. setaceus* from its close relative *A. officinalis* of ~9.66 million years ago, and *A. setaceus* underwent two rounds of whole-genome duplication. In addition, 762 specific gene families, 96 positively selected genes, and 76 resistance (R) genes were detected and functionally predicted in *A. setaceus*. These findings provide new knowledge about the characteristics and evolution of the *A. setaceus* genome, and will facilitate comparative genetic and genomic research on the genus *Asparagus*.

## Introduction

*Asparagus* L. is a monocot genus belonging to Asparagaceae (Asparagales) that comprises >200 species distributed widely in regions with an arid–subarid climate in the Old World^1–3^. This genus includes commercially important vegetable species, most prominently *A. officinalis*, and some species with great ornamental and/or medicinal value, such as *A. setaceus* and *A. cochinchinensis*. There are three subgenera in the *Asparagus* genus: *Asparagus*, *Myrsiphyllum*, and *Protasparagus*^2,4,5^. Within the *Asparagus* subgenus, all species are dioecious, whereas the species in the *Myrsiphyllum* and *Protasparagus* subgenera are hermaphroditic^2^. Although this genus has important commercial value, only the model dioecious plant *A. officinalis* has been extensively investigated, the including sequencing and assembly of a reference genome^6–11^. Other species, especially hermaphroditic species, are poorly investigated. The absence of a reference genome for hermaphroditic species has limited our understanding of the biology and evolution of the *Asparagus* genus.

**Fig. 1:**
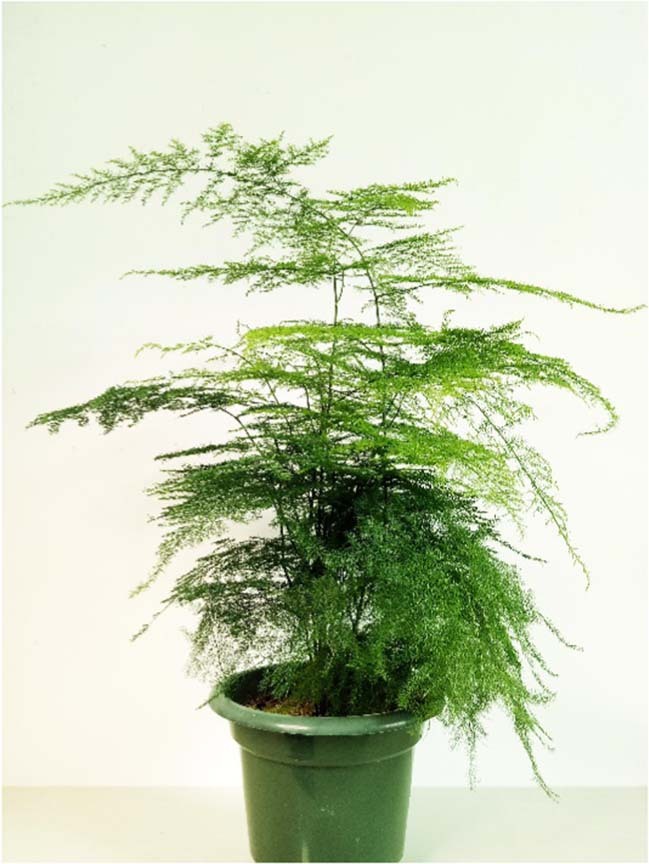
Image of an *A. setaceus* plant

Among hermaphroditic *Asparagus* species, *A. setaceus* (synonyms: *A. plumosus*, *Protasparagus plumosus*, and *P. setaceus*) is a scrambling perennial herb with needle-like fascicled cladodes^12^. It is a very popular ornamental plant because of its attractive traits of extremely feathery, soft leaves, and an elegant posture (Fig. 1). *A. setaceus* also has multiple uses in traditional oriental medicine^13^. As a wild relative species of the important vegetable *A. officinalis*, *A. setaceus* is resistant to purple spot disease caused by infection with *Stemphylium vesicarium*^14^ and rust disease caused by infection with *Puccinia asparagi*^5^, which are common pathogens of *A. officinalis*^15^. Investigating the mechanisms of agricultural characteristics related to pathogen resistance is potentially valuable for the molecular breeding of *A. officinalis*. Therefore, *A. setaceus* has high commercial and medicinal value, and is the subject of scientific research because of its properties. However, research on this species is limited. Only a few studies have explored the base chromosome number (2*n* = 2*x* = 20) and karyotype of *A. setaceus*^16^ and its genome size (~720 Mb)^17^, micropropagation^12^, chloroplast genome^18^, and phylogenetic relationships with other *Asparagus* species^19,20^. Genome sequence analysis can greatly promote molecular and genetic studies on this species, and the *A. officinalis*–*A. setaceus* genome pair provides a suitable model for the evolutionary analysis of the *Asparagus* genus.

In this study, we de novo assembled the genome of *A. setaceus* through a combination of sequencing strategies, including the use of Nanopore, Illumina, 10× Genomics, and Hi-C technology. Genome annotation, the characterization of genome features, evolutionary analysis, and resistance gene identification were performed based on the assembled genome sequences. Our results provide a foundation for further genome-wide studies on *A. setaceus* and will be useful for studying the evolution of the *Asparagus* genus.

## Results

### Genome sequencing and assembly

**Fig. 2:**
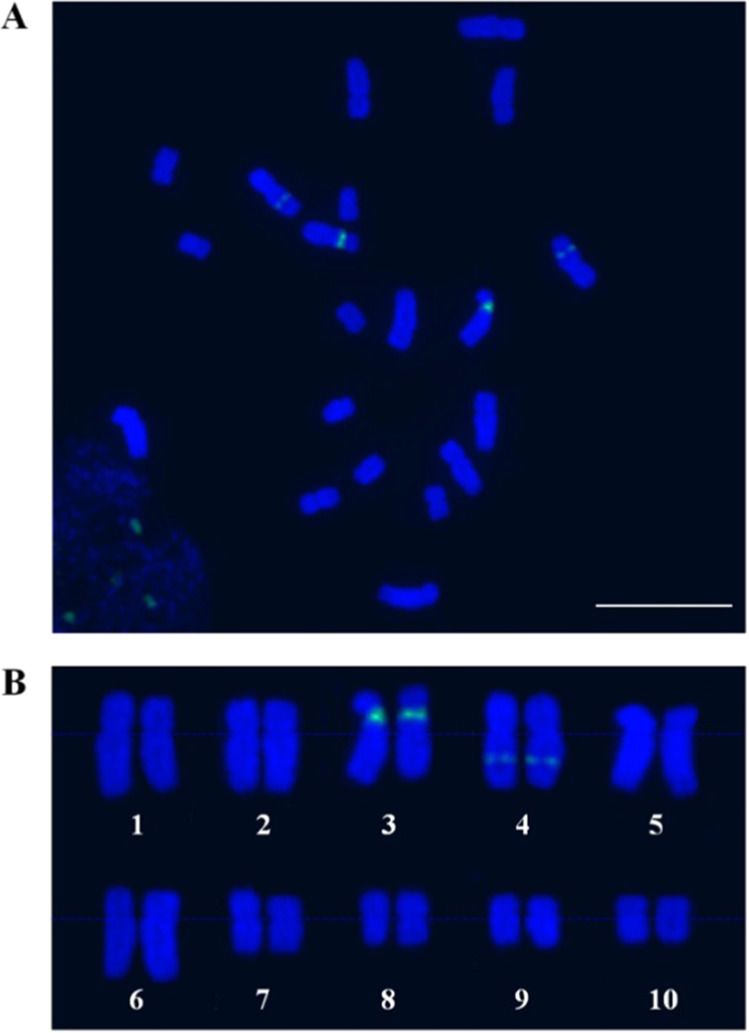
Cytogenetic investigation of *A. setaceus* chromosomes. **a** Cytological analysis of *A. setaceus* chromosomes in root tip cells. **b** Karyotyping of *A. setaceus* chromosomes based on 45 S rDNA FISH. The 45 S rDNA was labeled with Chroma Tide Alexa Fluor 488 (green), and the chromosomes were counterstained with DAPI (blue). Scale bar, 10 μm

A single plant *A. setaceus*, which has ten homologous pairs of chromosomes in diploid cells (Fig. 2), was used for genome sequencing. The analysis of the 17-mer frequency revealed high genome heterozygosity of 1.9% (Supplementary Fig. 1).

**Fig. 3:**
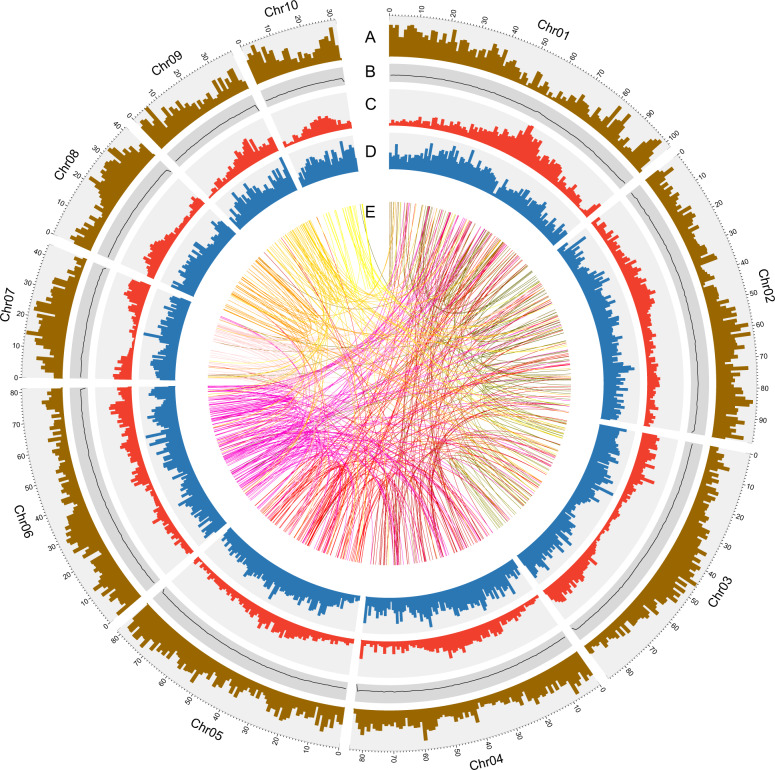
Characterization of the *A. setaceus* genome. Ten pseudochromosomes were ordered by size. A: Gene density (brown); B: GC content (black); C: density of *Gypsy* retrotransposable elements (red); D: density of *Copia* retrotransposable elements (blue); and E: relationship between syntenic blocks, as indicated by lines

For accurate assembly in this highly heterozygous plant, we sequenced the genome by utilizing a combination of Illumina, Nanopore, 10× Genomics, and Hi-C approaches, and assembled the sequences by using a series of methods. The sequencing and genome assembly workflow is shown in Supplementary Fig. 2a. We obtained a total of 112.52 Gb of Nanopore long reads (Supplementary Fig. 2b) corresponding to ~156.28× coverage of the ~720 Mb *A. setaceus* genome, as estimated using flow cytometry^17^. The Nanopore long reads were assembled into contigs by de novo methods. The primary contigs were adjusted with Illumina paired-end reads (84.63 Gb) and then employed for scaffold assembly using 10× Genomics data. The ~180 Gb of 10× Genomics sequencing reads included 2615 contigs grouped into 2061 scaffolds. After redundancy was removed, the assembled genome included 1393 scaffolds with an N50 length of 2.19 Mb. We further connected these scaffolds into superscaffolds by using Hi-C reads (~96 Gb, 133-fold coverage; Supplementary Fig. 2c). Ten of the largest superscaffolds exhibited a total length of 687.77 Mb and matched the ten *A. setaceus* chromosomes (Supplementary Table 1). The final assembly of the *A. setaceus* genome was 710.15 Mb in length, constituting 98.63% of the predicted genome size. Among the obtained sequences, 96.85% were anchored to the ten chromosomes (Fig. 3), whereas 22.38 Mb in 657 scaffolds remained unmapped. The sequencing and assembly information are summarized in Table 1.

**Table 1:** Statistics of the sequencing and assembly of the *A. setaceus* genome

	Number	Size	Sequence coverage (×)
Estimation of genome size		720.00 Mb	
Nanopore reads	7,729,366	112.52 Gb	156.28
Illumina reads	562,169,012	84.63 Gb	117.54
10× Genomics barcode reads	1,235,131,907	179.62 Gb	249.47
Hi-C reads	667,199,524	96.14 Gb	133.53
Scaffolds	1393	710.05 Mb	
Chromosome-anchored scaffolds	736	687.77 Mb	
Assembled genome size		710.15 Mb	
N50 of contigs		1.36 Mb	
N50 of scaffolds		2.19 Mb	

### Evaluation of the genome assembly

The completeness of this assembled genome was assessed using BUSCO analysis^21^. Approximately 90.0% of the plant orthologs were included in the assembled sequences (Supplementary Table 2). Furthermore, 89.85% of the transcriptome reads could be mapped to the assembled sequences. In the karyotype of *A. setaceus*, the first six pairs of chromosomes were clearly larger than the remaining chromosomes (Fig. 2), and the assembly results were consistent with this observation (Fig. 3). These results suggested a high accuracy and completeness of the genome assembly.

### Annotation of the *A. setaceus* genome assembly

We combined different strategies to identify protein-coding genes (Supplementary Fig. 2d). A total of 28,410 genes were identified in the *A. setaceus* genome (Supplementary Table 3). The average gene length was 6398 bp, and the mean exon number of each gene was 4.95 (Supplementary Table 4). Among these genes, 90.28% showed homology with known genes according to BLAST analysis (Supplementary Table 5). In addition, 2126 noncoding RNAs, including 388 microRNAs (miRNAs), 784 tRNAs, 273 rRNAs, and 681 small nuclear RNAs (snRNAs), were detected (Supplementary Table 6).

Complicated transposable element (TE) annotation showed that 64.43% of the *A. setaceus* genome assembly was comprised of TEs. Among these TEs, long terminal repeats were predominant, constituting ~42.51% of the assembled genome of *A. setaceus* (Supplementary Table 7). DNA transposons, long interspersed nuclear elements (LINEs), and short interspersed nuclear elements (SINEs) accounted for 4.12%, 2.90%, and 0.04% of the total assembly, respectively (Supplementary Table 7). Simple sequence repeats (SSRs) are another type of important tandemly repetitive sequences. We used MISA software to detect SSRs in the genome of *A. setaceus*. A total of 215,955 SSRs (i.e., 85,131 mono-, 103,002 di-, 20,878 tri-, 3683 tetra-, 1967 penta-, and 1294 hexa-nucleotide repeats) were detected (Supplementary Table 8). The total length of the SSR sequences was 8,914,237 bp, accounting for ~1.26% of the assembled *A. setaceus* genome. Thus, repetitive sequences, including TEs and SSRs, occupied 65.59% of the *A. setaceus* genome. The basic annotation information is listed in Table 2. The gene density, GC content, *Gypsy*, and *Copia* density mapped on the ten *A. setaceus* chromosomes are shown in a circos plot in Fig. 3.

**Table 2:** Annotation of the *A. setaceus* genome assembly

	Number	Size	Percentage
GC content			38.70%
Total TE sequences	997,186	457.56 Mb	64.43%
Total protein-coding genes	28,410		
Annotated protein-coding genes	25,649		90.28%
Average length per gene		6398 bp	
Average exons per gene	4.95		
Average length per intron		1343 bp	
Noncoding RNAs	2126		

### Evolutionary analysis

Evolutionary analysis was performed by comparing the *A. setaceus* genome with the genomes of 12 other representative plant species. These species included one other plant in the *Asparagus* genus (*A. officinalis*), one additional plant in the Asparagales order (*Phalaenopsis equestris*), three additional plants in the monocot clade (*Oryza sativa*, *Phoenix dactylifera*, and *Musa acuminate*), and seven other representative plants in the eudicot clade (*Amborella trichopoda*, *Solanum lycopersicum*, *Arabidopsis thaliana*, *Carica papaya*, *Populus trichocarpa*, *Spinacia oleracea*, and *Vitis vinifera*).

**Fig. 4:**
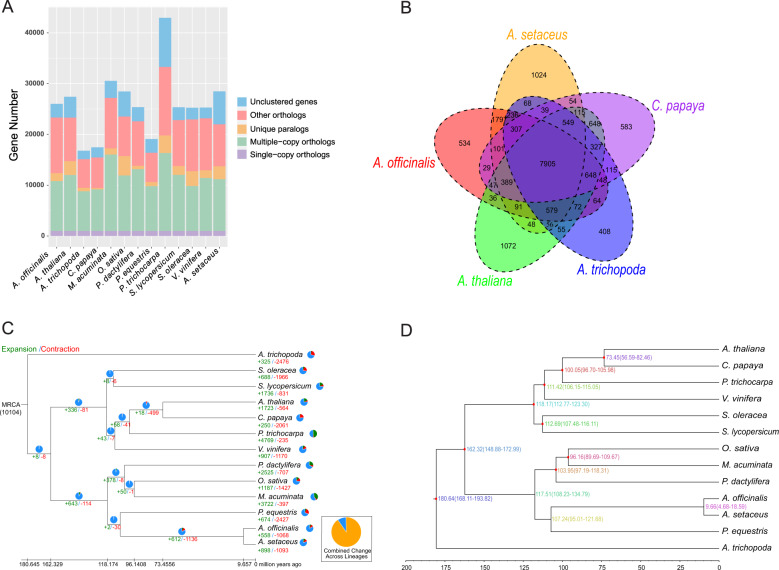
Gene family and phylogenetic analysis of *A. setaceus* and other representative plant genomes. **a** Clusters of orthologous and paralogous gene families in *A. setaceus* and 12 other sequenced plant genomes. **b** Venn diagram representing the distribution of shared gene families among *A. setaceus* and four other plants (*A. officinalis*, *A. thaliana*, *C. papaya*, and *A. trichopoda*). **c** CAFÉ-based estimates of gene family expansions and contractions. The green and red numbers indicate expanded and contracted gene families, respectively. Conserved gene families are indicated in blue in the pi charts. MRCA represents the most recent common ancestor. **d** Phylogenetic tree of *A. setaceus* and 12 other species based on a concatenated alignment of 1002 single-copy ortholog gene sets. The tree is rooted with *A. trichopoda* as the outgroup

OrthoMCL gene family clustering analysis revealed a total of 13,355 gene families consisting of 21,981 genes in the *A. setaceus* genome (Fig. 4a, Supplementary Table 9). OrthoMCL clustering recovered 1002 strictly single-copy ortholog gene sets among the 13 analyzed species. Ortholog analysis revealed that *A. setaceus*, *A. officinalis*, *C. papaya*, *A. thaliana*, and *A. trichopoda* shared a core set of 7905 gene families (Fig. 4b). Further large-scale analysis among *A. setaceus* and the 12 other selected species showed that 762 gene families specific to *A. setaceus* (Supplementary Table 9). These specific genes are mostly involved in heme binding, DNA binding, oxidoreductase activity, and iron and zinc ion binding (Supplementary Fig. 3).

Further gene family analysis revealed that 898 gene families were expanded in *A. setaceus*, whereas 1093 gene families were lost from the *A. setaceus* genome (Fig. 4c). In comparison with the close relative *A. officinalis*, which exhibits 558 expanded gene families and 1068 missing gene families, *A. setaceus* has gained more gene families. These expanded genes present diverse functions, such as binding, protein kinase activity, oxidoreductase activity, and transferase activity. As expected, a phylogenetic analysis showed that *A. setaceus* displayed a closer relationship to *A. officinalis* and phylogenetically diverged from the common ancestor ~9.66 million years ago (Mya), after the separation of Orchidaceae at 107.24 Mya (Fig. 4d).

**Fig. 5:**
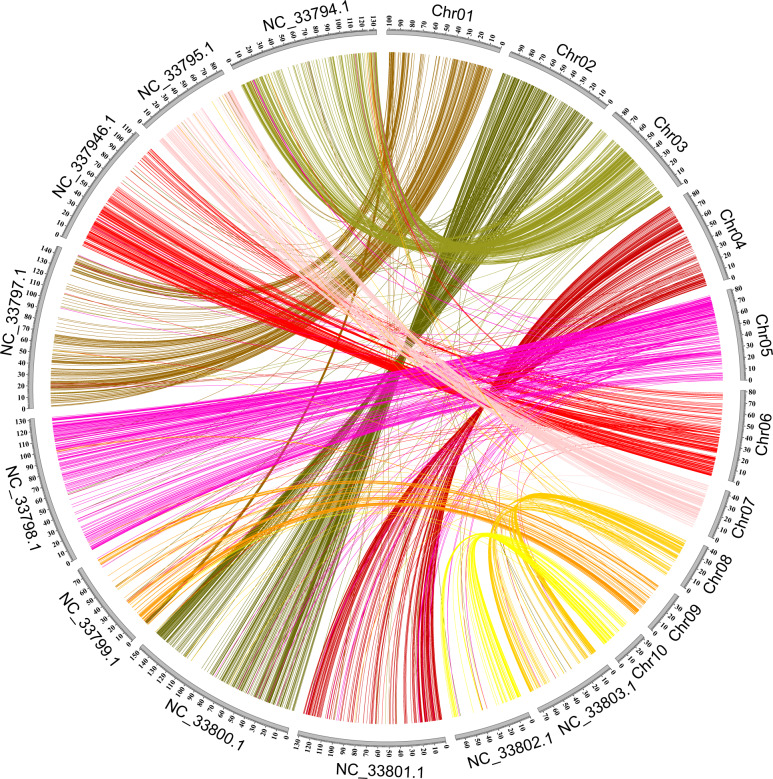
Macrosynteny analysis between the *A. setaceus* and *A. officinalis* chromosomes. Chr01–Chr10 represent the chromosomes of *A. setaceus*, while NC33794.1–NC33803.1 correspond to the chromosomes of *A. officinalis*

Synteny analysis was performed for the *A. setaceus* and *A. officinalis* genomes to understand the genome evolution of these two related species. High collinearity was observed between these two genomes (Fig. 5). The relationships between the chromosomes of *A. setaceus* and *A. officinalis* were illustrated based on the shared syntenic blocks. In general, each chromosome of *A. setaceus* corresponded to one chromosome of *A. officinalis*. For instance, *A. setaceus* Chr03 matched the sex chromosome of *A. officinalis* (Chr01, NC33794.1). In detail, 453 syntenic blocks containing more than three genes were identified from *A. setaceus* and *A. officinalis*. The largest synteny block containing 329 genes was found between *A. setaceus* chromosome 03 and the *A. officinalis* sex chromosome (01, NC33794.1). Furthermore, the visualization of synteny blocks revealed frequent interchromosomal rearrangement events between the chromosomes of *A. setaceus* and *A. officinalis* (Fig. 5). For example, most of the synteny blocks of *A. setaceus* Chr05 matched *A. officinalis* Chr05 (NC33798.1), but a few of them corresponded to *A. officinalis* Chr06 (NC33799.1), Chr07 (NC33800.1), Chr08 (NC33801.1), Chr09 (NC33802.1), or Chr10 (NC33803.1).

### Genome expansion in *A. setaceus*

**Fig. 6:**
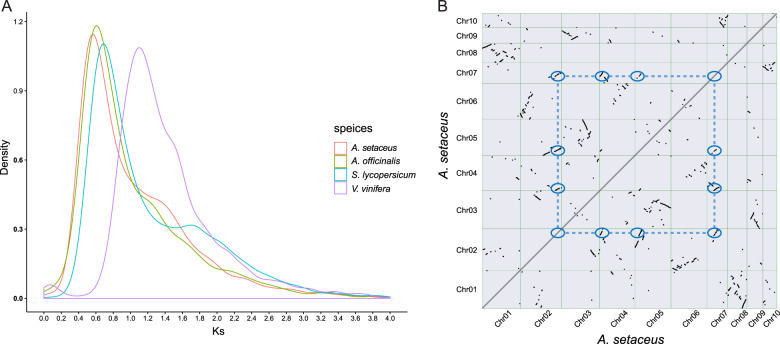
WGD analysis of the *A. setaceus* genome. **a** 4DTv distribution in *A. setaceus* and other representative plant species. **b** Dot plots of paralogs in the *A. setaceus* genome, illustrating the two rounds of WGD events

To investigate the genome expansion in *A. setaceus*, we analyzed whole-genome duplication (WGD) events. 4DTv and Ks values were estimated on the basis of the paralogous gene pairs in collinear regions detected in *A. setaceus* and three other representative plant species, *P. dactylifera*, *P. equestris*, and *V. vinifera*. The distribution of Ks or 4DTv values in *A. setaceus* showed two distinct peaks at Ks values of ~0.58 (4DTv ~0.18) and ~1.00 (4DTv ~0.45; Fig. 6a, Supplementary Fig. 4). The first peak was shared by *A. setaceus* and *A. officinalis*, and may correspond to the Asparagales-α event previously identified in the *A. officinalis* genome^11^. The second is predicted to be derived from a more ancient WGD, which was also found in the *A. officinalis* genome based on Ks analysis (Supplementary Fig. 4). Dot plots (Fig. 6b) are presented for the paralogs that evolved from the two rounds of WGD events in *A. setaceus* genome (4–4 diagonal relationships).

### Positively selected genes in *A. setaceus*

To detect positively selected genes in *A. setaceus*, we evaluated the Ka/Ks ratios of genes with only one copy by using *A. setaceus* as a predetermined (foreground) branch and *A. officinalis* as a background branch. We detected 96 genes that have probably experienced positive selection. GO enrichment revealed that a majority of these genes were involved in ATP binding, nucleic acid binding, oxidoreductase activity, and oxidation–reduction processes (Supplementary Table 10).

### Resistance R genes

The *A. setaceus* genome included 76 resistance (R) genes with nucleotide-binding sites (NBSs). These genes constituted ~0.27% of all *A. setaceus* genes. Among these genes, 73 resided on the chromosomes, and three genes were located on unmapped scaffolds. These R genes belonged to five groups: TIR-NBS, CC-NBS-LRR, NBS-LRR, NBS, and CC-NBS. NBS-LRR was the largest group, including a total of 29 genes (Supplementary Table 12).

## Discussion

*A. setaceus* is a popular ornamental plant species in many areas of the world. This plant species also has medicinal value. In addition, as a close relative of the important vegetable *A. officinalis*, comparative genetic and genomic studies on *A. setaceus* and *A. officinalis* are helpful for investigating the mechanisms of disease resistance-related agricultural traits, and the origin and evolution of the sex chromosomes of *A. officinalis*. However, studies on this species are very limited. In particular, molecular-level studies are almost nonexistent. A genome sequence could greatly promote studies on this species and contribute to the comparative analysis of related *Asparagus* species.

*A. setaceus* cv. ‘Pyramidalis’ shows a very high level of heterozygosity (1.9%) and a high content of repeats (64.32%). The assembly of such a highly heterozygous and repetitive genome is a challenging task^22,23^. Thus, we used a series of sequencing strategies, including the Nanopore, Illumina, 10× Genomics, and Hi-C sequencing platforms. We used Nanopore long reads for primary assembly, followed by assembly adjustment with highly accurate short reads. Then, 10× Genomics and Hi-C sequencing data were adopted for scaffold extension and superscaffold (chromosome) construction. By taking full advantage of these sequencing technologies, a chromosome-level genome assembly with high completeness and accuracy was obtained for *A. setaceus*. BUSCO assessment revealed that 90.0% of the complete BUSCOs could be found in the current assembled *A. setaceus* genome. This percentage was lower than those in *Osmanthus fragrans* (96.1%)^24^ and *Brassica oleracea* (96.77%)^25^, but higher than those in the genomes of some other species, such as *Ginkgo biloba* (73.95%)^26^ and *A. officinalis* (88.2%)^11^. Considering the high level of heterozygosity and repetitiveness of the genome, the current version represents a high-quality genome assembly of *A. setaceus*.

To guarantee the accuracy of the genome annotation, we integrated various methods to annotate protein-coding genes and used an integrated pipeline to analyze repetitive sequences. A vast majority of the genes in the *A. setaceus* genome were functionally annotated. Repetitive sequences, mainly consisting of TEs, constitute a major fraction of eukaryotic genomes and play vital roles in genome evolution^27,28^, chromosome rearrangement^29^, and gene regulation^30^. Repetitive sequences occupied 65.59% of the *A. setaceus* genome assembly; this percentage is very similar to that in the *A. officinalis* genome (69%).

Comparative genomics analysis showed high synteny and colinearity between the genomes of *A. setaceus* and *A. officinalis*. This observation is consistent with their close relationship. These two species diverged from their last common ancestor ~9.66 Mya. Thus, the transition from hermaphroditism to dioecy in *Asparagus* occurred <9.66 Mya, and the sex chromosome of *A. officinalis* evolved from the ancestral autosome recently. The results agree with previous findings showing that the cytologically homomorphic X and Y sex chromosomes of *A. officinalis* are very young^19,31,32^. However, the accurate timing of the sex chromosome origin will require further analysis. We found that the *A. officinalis* sex chromosome and *A. setaceus* Chr03 shared a common ancestral chromosome. Further detailed comparative analyses of these two chromosomes would increase our knowledge of the evolution of the sex chromosome of *A. officinalis*.

It has been demonstrated that WGD events contribute greatly to the evolution of genomes and genes. Recent evidence has revealed that different plant lineages have experienced distinct WGD events. For example, the grape genome did not experience WGD after the γ-event shared by eudicot plants that took place ~140 Mya, whereas the tea genome underwent two additional rounds of WGD^33^. The monocot species share a common WGD event, after which different species experienced lineage-specific WGD events^34^. A previous study revealed that the *A. officinalis* genome underwent at least two ancient WGDs before the divergence of *A. officinalis* and other *Asparagus* species^11^. The high synteny and collinearity of the genomes of *A. setaceus* and *A. officinalis* are in accord with the likely possibility that the two genomes experienced the same WGD events. In this study, the Ks and 4DTv distribution analysis revealed two distinct peaks, which likely correspond to the two rounds of WGD detected in *A. officinalis*^11^. The most ancient WGD event was not very clear in the *A. officinalis* genome based on the current analysis. This may be because of the gene loss process following the WGD events in the *A. officinalis* genome. The WGD events and subsequent diploidization have contributed greatly to the current genome structure of *A. setaceus*.

As a wild relative species of garden asparagus, *A. setaceus* is resistant to some common diseases caused by plant pathogens in *A. officinalis*^5,14,15^. In plants, R genes are usually involved in defense mechanisms against infections caused by a majority of specialized plant pathogens^35^. Thus, examining the resistance genes of *A. setaceus* is helpful for the further molecular breeding of *A. officinalis*. Most of the extensively investigated plant R genes contain NBSs^35^. The *A. officinalis* genome contains 49 different NBS R genes^36^. In this study, we identified 76 non-redundant R genes in the *A. setaceus* genome, which was greater than the number in *A. officinalis*. Functional studies on these genes would improve our understanding of *A. setaceus* defense mechanisms and provide a basis for the molecular breeding of *A. officinalis*.

## Conclusion

A chromosome-scale reference genome of *A. setaceus* (~710.15 Mb) was generated by combining the Nanopore, Illumina, 10× Genomics, and Hi-C sequencing platforms. A total of 28,410 genes were identified. Among these genes, 90.22% were annotated. Repetitive sequences occupied 65.59% of the genome. The divergence between *A. setaceus* and *A. officinalis* is estimated to have occurred ~9.66 Mya. Genome evolution analysis provided evidence supporting two rounds of WGD events. The identified genomic features of *A. setaceus*, including gene families, syntenic blocks, WGD events, and genome-specific genes, provide rich data for comparative genomic studies in plants, especially for studying species in the same genus. The divergence time and synteny analysis between *A. setaceus* and *A. officinalis* will contribute to studies on the evolution of the sex chromosome of *A. officinalis* and the *Asparagus* genus.

## Materials and methods

### Molecular karyotype analysis of *A. setaceus*

A plant of *A. setaceus* cv. ‘Pyramidalis’ cultivated in the glasshouse of Henan Normal University was used in this study. The preparation of mitotic metaphase spreads, fluorescence in situ hybridization (FISH), and molecular karyotype analysis were performed as previously described^37^.

### Genome sequencing

Total DNA was isolated from young fascicled cladodes and stems by using the CTAB method to construct Nanopore and Illumina libraries. For each Nanopore library, the genomic DNA was fractionated (10–50 kb) with BluePippin (Sage Science, Beverly, MA), repaired, A tailed, adaptor ligated, and used for library construction in accordance with the Nanopore library construction protocol. A total of 67 libraries were generated and sequenced on the GridION X5 sequencer platform (Oxford Nanopore Technologies, UK) at the Nextomics Biosciences Company (Wuhan, China).

### 10× Genomics linked read sequencing

High-molecular weight DNA extraction, indexing, and barcoding were performed in accordance with the standard protocols provided by 10× Genomics. Approximately 1 ng of sample DNA was used for GEM generation, and 16 bp barcodes were used for the labeling of droplets. After the GEM reactions were thermally amplified, the droplets were fractured, and the intermediate DNA library was purified. Then, the DNA was sheared into 500 bp fragments to construct libraries. Sequencing was performed by using an Illumina HiSeq X Ten sequencer to generate linked reads. The long DNA molecules contained many short reads sharing the same barcode.

### Hi-C sequencing

Hi-C sequencing data were generated to obtain physical scaffolds for genome assembly as previously described^38^. Briefly, fresh spears were harvested, cut into small sections, and immersed in 2% formaldehyde for 15 min for crosslinking. Thereafter, the materials were crushed into a fine powder and used for the isolation of nuclei. The isolated nuclei were purified, digested with *Dpn* II, blunt-end-repaired, and tagged with biotin. Then, the DNA was religated with the T4 DNA ligation enzyme. After proteinase K digestion and the reversion of formaldehyde crosslinking, biotin-containing DNA fragments were captured and used for the construction of the Hi-C library. The final libraries were sequenced by using an Illumina HiSeq X ten sequencer.

### RNA-seq

Total RNA was isolated separately from leaves, stems, and flowers of the same *A. setaceus* individual by using a QIAGEN RNeasy plant mini kit (QIAGEN, Hilden, Germany). Thereafter, RNA-seq libraries were constructed with a TruSeq RNA library preparation kit (Illumina), and PE150 sequencing was carried out on the HiSeq X ten platform. A total of 7.8 Gb, 7.1 Gb, and 8.5 Gb of sequences were generated from the three sample types. In addition, full-length transcriptome sequencing was conducted for mixed samples by using the PacBio Sequel platform, obtaining an additional 14 Gb of data.

### Heterozygosity estimation

The heterozygosity of *A. setaceus* was estimated via K-mer frequency analysis by using Illumina sequencing data^39^ in accordance with previously described methods^24^.

### Genome assembly

Oxford Nanopore sequencing data were filtered (mean_qscore > 7) and then employed for genome assembly by using the complete Canu pipeline with default parameters^40^. The paired-end Illumina reads were mapped to the assembly to improve its accuracy for base-pair correction with BWA MEM^41^ and Pilon^42^.

The scaffolds were extended with 10× Genomics data by using ARKS^43^ with the following parameters: “-c 5 -j 0.55 -m 50-10000 -k 30 -r 0.05 -e 3000 -z 500 -d 0”, and LINKS^44^ with the following parameters: “-l 5 -a 0.9 -z 500”. Thereafter, redundancy in the assembly was removed using redundans 0-13c^45^ with the following parameters: “–identity 0.7 –overlap 0.7”.

The Hi-C sequencing data were used for the scaffolding of the preliminary assemblies and to increase the contiguity of the assembly at the chromosome level. The cleaned paired-end reads generated by the Illumina HiSeq platform from the Hi-C library were aligned to the assemblies by using Bowtie2 (version 2.3.2)^46^. After the map position and orientation of the unique mapped reads were considered, the validated read pairs were filtered. Then, LACHESIS software^47^, which applies a hierarchical agglomerative clustering strategy, was used for chromosome-level scaffolding by clustering, ordering, and orienting the previous assemblies based on genomic proximity information between Hi-C read pairs. Finally, the adjacent anchored scaffolds were connected using 100 bp Ns to form ten superscaffolds corresponding to ten chromosomes.

### Gene and repetitive sequence annotation

Protein-coding genes were identified using strategies that combined de novo gene prediction, experimental evidence obtained from transcriptomic data, and homology-based methods. For homology prediction, GeMoMa^48^ was used with a protein sequence from *A. officinalis*, a relative of *A. setaceus*. For RNA-seq-based prediction, PASA^49^ was used on the basis of the assembled RNA-seq unigenes. Augustus^50^ was used for de novo prediction. Then, genes identified by these methods were integrated with EVM^51^. Then, the sequences of the predicted genes were searched against the commonly used SwissProt, GO, KEGG, KOG, Nr, and InterPro databases for annotation.

For the annotation of noncoding RNAs, tRNAscan-SE software^52^ was used to predict the tRNAs with eukaryotic parameters. miRNAs, rRNAs, and snRNAs were detected using Infernal cmscan^53^ to search the Rfam database^54^. The rRNAs and the corresponding subunits were annotated with RNAmmer v1.2^55^.

Repeat annotation was conducted using RepeatMasker based on a custom library produced using de novo-based and homology-based strategies. The de novo prediction of repeats was carried out by using RepeatModeler. A homology-based detection procedure was performed using a conserved BLASTN search in Repbase^56^. The consensus families generated by RepeatModeler and repeat sequences with similarity in Repbase were merged as a database to analyze the *A. setaceus* genome by using RepeatMasker. The genome annotation pipeline is presented in Supplementary Fig. 2d.

### Gene family analysis

The protein data of some representative plant species, including *A. thaliana*, *A. setaceus*, *A. officinalis*, *A. trichopoda*, *C. papaya*, *M. acuminate*, *O. sativa*, *P. dactylifera*, *P. equestris*, *P. trichocarpa*, *S. lycopersicum*, *S. oleracea*, and *V. vinifera*, were retrieved from the NCBI database and used for gene family clustering. All protein sequences were pooled and clustered into different kinds of homologs by using the software OrthoMCL with default parameter settings^57^.

### Phylogenetic tree reconstruction and divergence time prediction

A total of 1002 single-copy genes shared by the analyzed genomes were used for subsequent phylogenetic tree building and divergence time evaluation. The selected protein sequences were concatenated and subjected to multiple alignments by using MAFFT^58^, and the less regions were filtered using Gblocks^59^. Then, a phylogenetic tree was constructed using RAxML^60^, and *A. trichopoda* was used as the root. The divergence time was estimated using MCMCtree, which was incorporated in the PAML package^61^. The expansion and contraction of the gene family were analyzed with CAFE (v1.6)^62^.

### Detection of polyploidization events

To detect the polyploidization events in the *A. setaceus* genome, the protein sequences from *A. setaceus* were intercompared by using BLASTP (*E*-value < 1e−05) to identify the conserved paralogs. Protein sequences of *P. dactylifera*, *V. vinifera*, and *P. equestris* were also analyzed, and used for comparison. Then, the WGD events of each species were estimated on the basis of the 4DTv and Ks distributions.

### Positively selected gene analysis

To detect the positively selected genes in *A. setaceus*, the single-copy genes of *A. setaceus* and the closely related species *A. officinalis* were aligned using MUSCLE^63^. Positive selection sites were detected with *A. setaceus* as a predetermined branch by using Codeml software (part of the PAML program package) with a branch-site model. The positively selected genes were annotated by GO and KEGG analyses.

### Identification of resistance (R) genes

To identify R genes, the *A. setaceus* genome was queried with HMM search by using the HMM profile of the NB-ARC domain (Pfam accession number: PF00931). Then, the NBS domain of the candidate genes was confirmed using the NCBI Conserved Domain Database (CDD)^64^ and the Pfam database^65^. The genes without an NBS domain were removed. The confirmed genes belonging to different groups were classified based on the conserved domains that they encoded using the CDD and Pfam databases.

## Supplementary information

Supplemental Table 1-9 and Supplemental Fig. 1-Fig. 4

Supplemental Table 10

Supplemental Table 11

## Data Availability

Raw data from this study were deposited in the NCBI SRA (Sequence Read Archive) database under the Bioproject ID: PRJNA564485. The genome sequence data (Nanopore, Illumina, 10× Genomics, and Hi-C data) are available under accession numbers SRR10176977, SRR10177257, SRR10176978, SRR10187020, and SRR10187021. Transcriptome data are available under accession numbers SRR10177390, SRR10177391, SRR10186988, and SRR10187001. The assembled genome sequences have been deposited at DDBJ/ENA/GenBank under the accession WHSE00000000. Gene models are available at Dryad (10.5061/dryad.1c59zw3rm).
