# Supplementary material for: Chromosome-level genome assembly, annotation and evolutionary analysis of the ornamental plant Asparagus setaceus
Source: Hortic Res. 2020 Apr 1;7:48. doi: 10.1038/s41438-020-0271-y (PMC7109074; doi:10.1038/s41438-020-0271-y)
Supplement: Supplementary file 1 — Supplemental Table 1-9 and Supplemental Fig. 1-Fig. 4 [file 41438_2020_271_MOESM1_ESM.docx]

**Supplemental Table 1 Statistics of the genome assembly of *A. setaceus*.**

| **Chr** | **Size** | **Scaf Num** |
| --- | --- | --- |
| Chr01 | 101,987,859 | 82 |
| Chr02 | 97,682,252 | 106 |
| Chr03 | 86,532,102 | 123 |
| Chr04 | 84,132,103 | 83 |
| Chr05 | 81,519,504 | 55 |
| Chr06 | 81,334,733 | 92 |
| Chr07 | 43,662,928 | 26 |
| Chr08 | 41,358,998 | 68 |
| Chr09 | 38,082,155 | 56 |
| Chr10 | 31,474,758 | 45 |
| Unplaced | 22,384,395 | 657 |

**Supplemental Table 2 Quality assessment of the assembled genome of *A. setaceus* using BUSCO.**

| **Type** | **Number** | **Percent (%)** |
| --- | --- | --- |
| Complete BUSCOs (C) | 1,237 | 90.0 |
| Complete and single-copy BUSCOs (S) | 1,159 | 84.3 |
| Complete and duplicated BUSCOs (D) | 78 | 5.7 |
| Fragmented BUSCOs (F) | 22 | 1.6 |
| Missing BUSCOs (M) | 116 | 8.4 |
| Total BUSCO groups searched | 1,375 | 100 |

**Supplemental Table 3 Summary statistics of annotated genes in *A. setaceus* genome.**

| **Gene set** | | **Total number of gene** | **Average transcript length(bp)** | **Average CDS length(bp)** | **Average exons number per gene** | **Average exons length(bp)** | **Average intro length(bp)** |
| --- | --- | --- | --- | --- | --- | --- | --- |
| De novo | AUGUSTUS | 31,198 | 5,978.16 | 1,075.46 | 5.05 | 212.9 | 1,210.13 |
|  | *A.offinalis* | 29,190 | 7,049.83 | 1,191.85 | 5.26 | 226.42 | 1,373.89 |
| Homology | *A.thaliana* | 20,752 | 7,783.24 | 1,272.22 | 5.68 | 223.99 | 1,391.29 |
|  | *E.guineensis* | 22,944 | 7,354.36 | 1,278.50 | 5.54 | 230.59 | 1,336.98 |
|  | *O.sativa* | 22,259 | 7,650.11 | 1,302.72 | 5.56 | 234.40 | 1,392.71 |
|  | *Z.mays* | 24,097 | 7,315.89 | 1,246.70 | 5.39 | 231.31 | 1,382.59 |
| PASA | Transdecoder | 14,627 | 7,843.14 | 1,285.69 | 6.15 | 209.04 | 1,273.21 |
| **Final set** | **EVM** | **28,410** | **6,397.81** | **1,086.38** | **4.95** | **219.30** | **1,343.37** |

**Supplemental Table 4 Summary statistics of the functional genes of *A. setaceus* and other model plant species.**

| **Species** | **Total number of gene** | **Average transcript length(bp)** | **Average CDS length(bp)** | **Average exon number per gene(bp)** | **Average exon length(bp)** | **Average intron length(bp)** |
| --- | --- | --- | --- | --- | --- | --- |
| *A.setaceus* | 28,410 | 6,397.81 | 1,086.38 | 4.95 | 219.30 | 1,343.37 |
| *A.offinalis* | 26,460 | 6,553.32 | 1,217.62 | 5.49 | 221.63 | 1,187.29 |
| *A.thaliana* | 27,411 | 1,856.41 | 1,205.11 | 5.09 | 236.78 | 159.26 |
| *E.guineensis* | 26,258 | 7,842.84 | 1,322.93 | 5.53 | 239.25 | 1439.43 |
| *O.sativa* | 28,577 | 3,111.11 | 1,338.30 | 5.05 | 264.96 | 437.64 |
| *Z.mays* | 37,262 | 4,130.55 | 1,180.58 | 4.76 | 248.26 | 785.54 |

**Supplemental Table 5 Annotation of the protein-coding genes of *A. setaceus*.**

|  | | **Type** | **Number** | **Percent (%)** |
| --- | --- | --- | --- | --- |
| Annotation | Swissprot | | 20,059 | 70.61 |
|  | KEGG | | 10,364 | 36.48 |
|  | KOG | | 13,654 | 48.06 |
|  | GO | | 11,762 | 41.40 |
|  | NR | | 25,442 | 89.55 |
| Total | Annotated | | 25,649 | 90.28 |
|  | Gene | | 28,410 |  |

**Supplemental Table 6 Identification of non-coding genes in *A. setaceus* genome.**

| **Type** | **Copy Number** | **Average Length (bp)** | **Total Length (bp)** | **Percentage (%) of Genome** |
| --- | --- | --- | --- | --- |
| rRNA | 273 | 607.05 | 165,726 | 0.012964 |
| 18S | 6 | 2,538.33 | 15,230 | 0.001191 |
| 28S | 17 | 7,171.47 | 121,915 | 0.009537 |
| 5.8S | 13 | 151.46 | 1,969 | 0.000154 |
| 5S | 237 | 112.29 | 26,612 | 0.002082 |
| snRNA | 681 | 112.67 | 76,728 | 0.006002 |
| CD-box | 453 | 97.74 | 44,276 | 0.003464 |
| HACA-box | 70 | 136.13 | 9,529 | 0.000745 |
| splicing | 158 | 145.08 | 22,923 | 0.001793 |
| miRNA | 388 | 147.28 | 57,144 | 0.004470 |
| tRNA | 784 | 74. 40 | 58,331 | 0.004563 |

**Supplemental Table 7 Summary statistics of the annotated transposable elements (TE) in the *A. setaceus* genome.**

| **Type** | **Number** | **Length (bp)** | **Percentage (%) of Genome** |
| --- | --- | --- | --- |
| Retrotransposon | 414,211 | 322,798,267 | 45.45 |
| LTR elements | 383,439 | 301,884,502 | 42.51 |
| Ty1/*Copia* | 87,525 | 57,350,547 | 8.08 |
| Ty3/*Gypsy* | 138,141 | 138,459,677 | 19.50 |
| LINEs | 28,578 | 20,599,454 | 2.90 |
| SINEs | 2,194 | 314,311 | 0.04 |
| DNA transposons | 139,954 | 29,274,477 | 4.12 |
| Unknown | 443,021 | 105,483,656 | 14.85 |
| Total | 997,186 | 457,556,400 | 64.43 |

**Supplemental Table 8 Specific statistics of the annotated SSRs in the *A*. *setaceus* genome.**

| **Type** | **Unit size**  **(repeat number)** | **Number** |
| --- | --- | --- |
| p1 | 1(>=10) | 85,131 |
| p2 | 2(>=6) | 103,002 |
| p3 | 3(>=5) | 20,878 |
| p4 | 4(>=5) | 3,683 |
| p5 | 5(>=5) | 1,967 |
| P6 | 6(>=5) | 1,294 |

**Supplemental Table 9 Summary statistics of the gene families of *A. setaceus* and other model plant species.**

| **Species** | **Gene number** | **Gene number in families** | **Unclustered gene number** | **Family number** | **Unique family number** | **Average gene number per family** |
| --- | --- | --- | --- | --- | --- | --- |
| *A.setaceus* | 28,410 | 21,981 | 6,429 | 13,355 | 761 | 1.65 |
| *A.offinalis* | 26,020 | 23,348 | 2,672 | 12,883 | 349 | 1.81 |
| *A.thaliana* | 27,417 | 23,311 | 4,106 | 12,637 | 712 | 1.84 |
| *A.trichopoda* | 16,795 | 15,132 | 1,663 | 11,476 | 217 | 1.32 |
| *C.papaya* | 17,458 | 15,475 | 1,983 | 11,904 | 79 | 1.30 |
| *M.acuminata* | 30,539 | 27,193 | 3,346 | 12,622 | 504 | 2.15 |
| *O.sativa* | 28,462 | 23,545 | 4,917 | 12,397 | 980 | 1.90 |
| *P.dactylifera* | 25,366 | 22,587 | 2,779 | 12,494 | 257 | 1.81 |
| *P.equestris* | 19,089 | 16,395 | 2,694 | 11,075 | 288 | 1.48 |
| *P.trichocarpa* | 42,950 | 33,288 | 9,662 | 14,253 | 1,132 | 2.34 |
| *S.lycopersicum* | 25,345 | 22,831 | 2,514 | 12,687 | 410 | 1.80 |
| *S.oleracea* | 25,242 | 22,967 | 2,275 | 12,252 | 606 | 1.87 |
| *V.vinifera* | 25,290 | 23,188 | 2,102 | 13,291 | 360 | 1.74 |


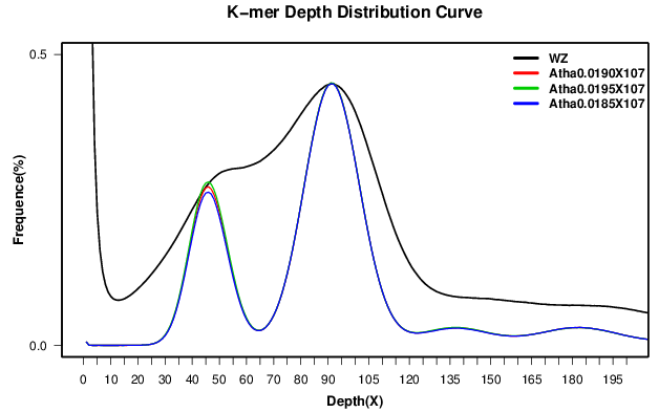


**Supplemental Fig. 1: The distribution of *A*. *setaceus* 17-mers.** The main peak is 92. Secondary peak is 45. The heterozygosity is consistent with the green line. Atha0.0190X107 means that the depth of the simulated Illumina PE reads of *Arabidopsis thaliana* is 107 with the expected heterozygosity of 1.90 %.


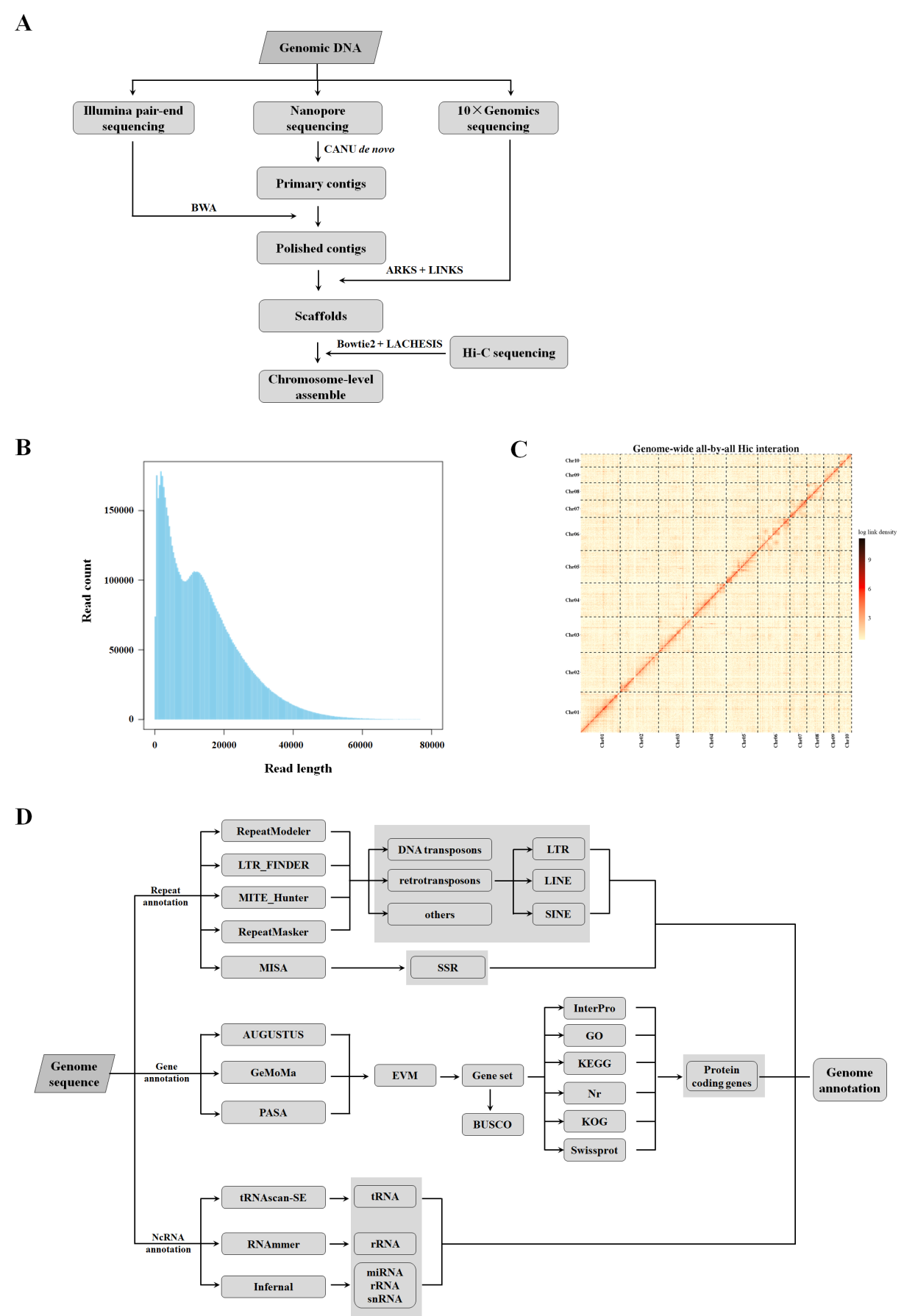


**Supplemental Fig. 2: Pipelines of genome assembly and annotation.**

A. The flow chart of the genome assembly of *A. setaceus*. B. Distribution of Nanopore read length. C. Hi-C heat map of the *A. setaceus* genome showing contact information. D. Workflow for the gene annotation pipeline.


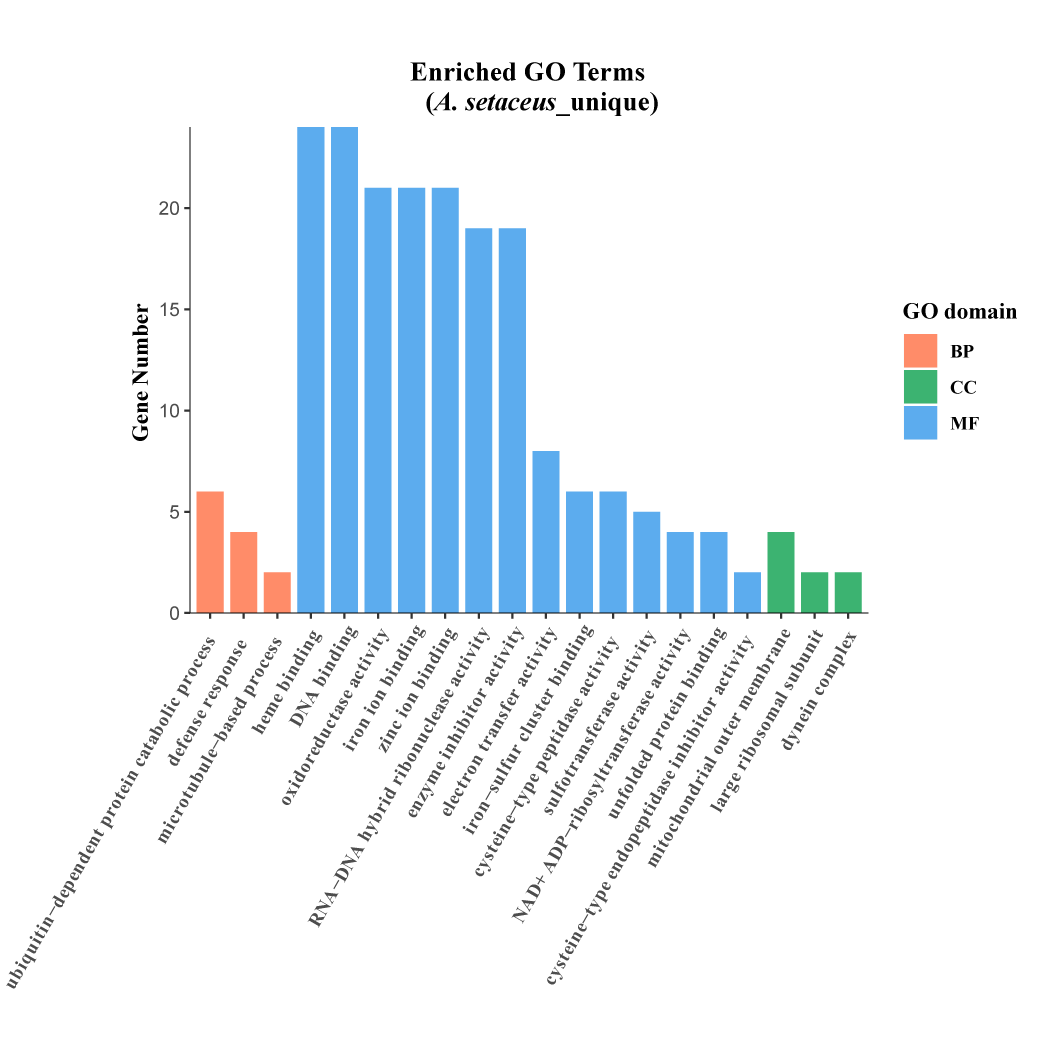


**Supplemental Fig. 3: Enriched GO terms of *A. setaceus* specific genes.**

**
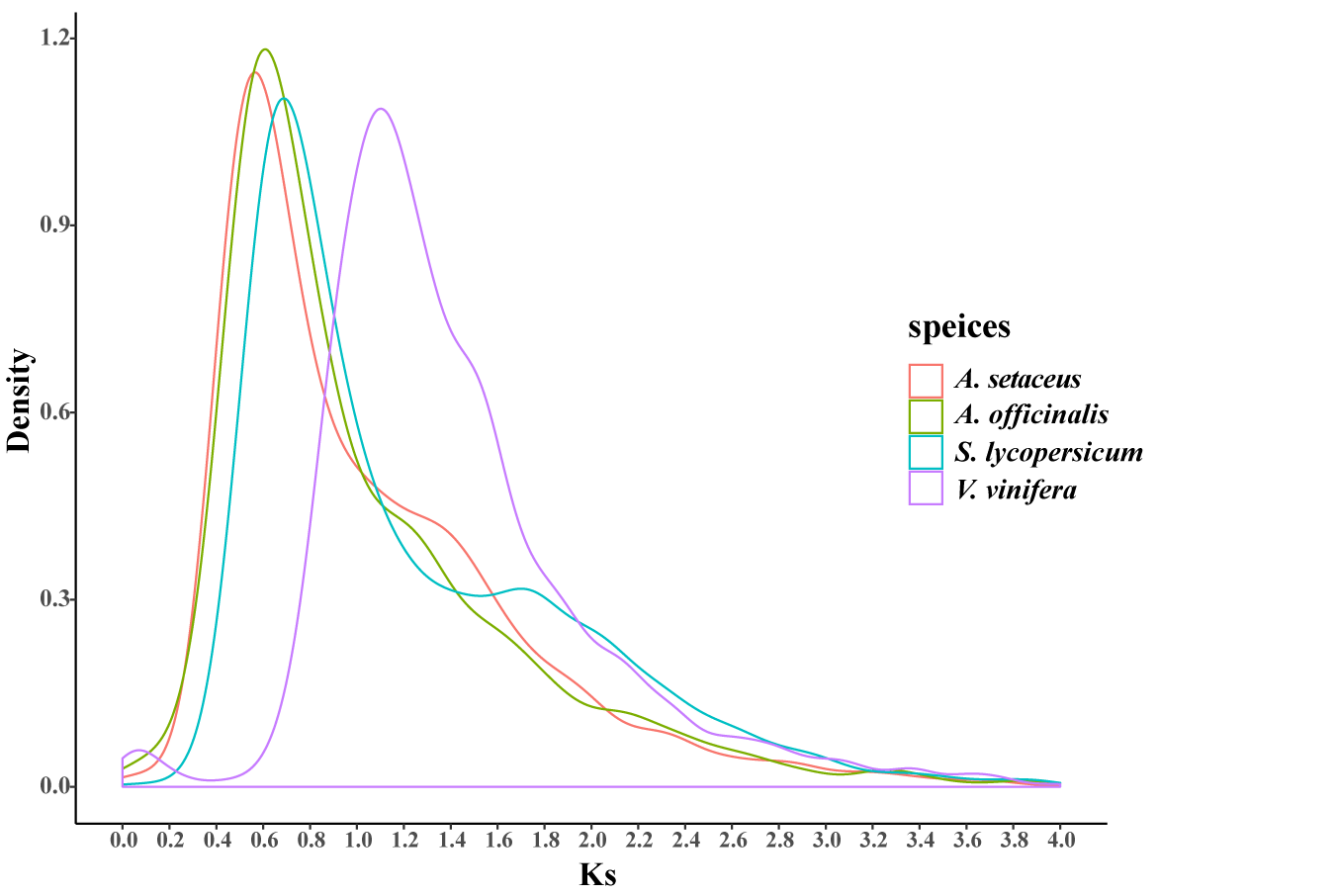
**

**Supplemental Fig. 4: Ks distribution of WGD in *A. setaceus* and other selected species.**
